# Long Noncoding RNAs in the Metastasis of Oral Squamous Cell Carcinoma

**DOI:** 10.3389/fonc.2020.616717

**Published:** 2021-01-15

**Authors:** Yuming Xu, Erhui Jiang, Zhe Shao, Zhengjun Shang

**Affiliations:** The State Key Laboratory Breeding Base of Basic Science of Stomatology (Hubei-MOST) & Key Laboratory of Oral Biomedicine Ministry of Education, School & Hospital of Stomatology, Wuhan University, Wuhan, China

**Keywords:** lncRNAs, cancer-associated fibroblasts, exosomes, oral squamous cell carcinoma, tumor microenvironment, metastasis

## Abstract

Oral squamous cell carcinoma (OSCC) is a common malignant tumor worldwide. Metastasis is the main cause of the death of OSCC patients. Long noncoding RNAs (lncRNAs), one of the key factors affecting OSCC metastasis, are a subtype of RNA with a length of more than 200 nucleotides that has little or no coding potential. In recent years, the important role played by lncRNAs in biological processes, such as chromatin modification, transcription regulation, RNA stability regulation, and mRNA translation, has been gradually revealed. More and more studies have shown that lncRNAs can regulate the metastasis of various tumors including OSCC at epigenetic, transcriptional, and post-transcriptional levels. In this review, we mainly discussed the role and possible mechanisms of lncRNAs in OSCC metastasis. Most lncRNAs act as oncogenes and only a few lncRNAs have been shown to inhibit OSCC metastasis. Besides, we briefly introduced the research status of cancer-associated fibroblasts-related lncRNAs in OSCC metastasis. Finally, we discussed the research prospects of lncRNAs-mediated crosstalk between OSCC cells and the tumor microenvironment in OSCC metastasis, especially the potential research value of exosomes and lymphangiogenesis. In general, lncRNAs are expected to be used for screening, treatment, and prognosis monitoring of OSCC metastasis, but more work is still required to better understand the biological function of lncRNAs.

## Introduction

Oral squamous cell carcinoma (OSCC) is the most common type of head and neck squamous cell carcinoma (HNSCC) which ranks sixth among cancers worldwide (1). The initiation and progression of OSCC are the results of the combined action of various factors, and tobacco, alcohol, and human papillomavirus (HPV) infection are considered as the main causes of OSCC (2). Surgical resection is an effective method to treat OSCC, and the surgical cure rate of patients with early-stage OSCC can reach more than 80% (3). However, the overall prognosis of patients with OSCC is poor, with a 5-year survival rate of only about 60% (4, 5). Metastasis is the major factor leading to the low survival rate of OSCC patients, and the 5-year survival rate of OSCC patients with metastasis is only about 40% (5). Notably, about 40% of OSCC patients may have metastasis (5). In addition, for non-metastatic patients, local resection of the primary tumor can achieve an ideal therapeutic effect, while metastatic patients generally need neck lymph node dissection, which not only increases the risk of surgery but also often causes postoperative dysfunction and maxillofacial deformities (6, 7). Whereas, it is still difficult to accurately determine whether OSCC patients have metastasis before surgery, which may lead to over-medical treatment (performing neck dissection for patients without lymph node metastasis) or insufficient treatment (patients with lymph node metastasis did not undergo neck dissection). Therefore, finding effective methods to screen, block, and treat OSCC metastasis is a top priority in OSCC research.

The metastasis of OSCC has two characteristics (5, 8): on the one hand, the metastasis rate of OSCC occurring in diverse anatomical parts is different, which is about 40% in the buccal and soft palate, but less than 30% in the tongue and floor of mouth; on the other hand, OSCC metastasis mainly manifests as regional cervical lymph node metastasis, distant metastasis is rare. Although the mechanisms behind this phenomenon are still unclear, current research indicated that enhanced migration and invasion ability of tumor cells (9) and lymphangiogenesis (10) may be critical factors that promote OSCC metastasis. Todorović et al. found that the expression levels of plakoglobin and desmoglein 2 (both are involved in maintaining intercellular adhesion) in squamous carcinoma cells derived from the top of the oral cavity are significantly lower than those in squamous carcinoma cells derived from the tongue, which is helpful for the migration and invasion of cells, and may be used to explain why the metastasis rate of OSCC in the soft palate is higher than that in the tongue (9). By analyzing the lymphatic vessel density in paraffin-embedded OSCC tissues, Sasahira et al. discovered that high lymphatic vessel density is closely related to lymph node metastasis and poor prognosis, which suggests that lymphangiogenesis may play a key role in the metastasis of OSCC (10). Unfortunately, scholars still have a limited understanding of the mechanisms of OSCC migration, invasion, and lymphangiogenesis, and have yet to find effective biomarkers.

Long noncoding RNAs (lncRNAs) are a subtype of RNA with a length of more than 200 nucleotides that has little or no coding potential (11). There are large amounts of lncRNAs in human cells that 68% of the human genome transcripts can be classified as lncRNAs (12). The expression of lncRNAs has certain tissue specificity (13), which suggests that they may play an important role in the biological processes of cells. Studies have confirmed that lncRNAs can interact with biological macromolecules including chromatin, protein, and RNA (14). In addition, many lncRNAs have been found to be abnormally expressed in various cancers, such as liver cancer (15), breast cancer (16), bladder cancer (17), and colorectal cancer (18), and regulate tumor cell metastasis through different mechanisms. In studies related to OSCC, lncRNAs were elucidated to be involved in tumor initiation (19) and prognosis (20). Furthermore, lncRNAs, such as MALAT1 (metastasis associated lung adenocarcinoma transcript 1) (21), UCA1 (urothelial cancer-associated 1) (22), AFAP1-AS1 (actin filament associated protein 1 antisense RNA 1) (23) and MEG3 (maternally expressed gene 3) (24) have been shown to participate in the regulation of OSCC migration, invasion, and metastasis. These studies have demonstrated the potential of lncRNAs for screening, treatment, and prognostic monitoring of OSCC metastasis. Therefore, in this paper, we review the role and possible mechanisms of lncRNAs in OSCC metastasis. In particular, we introduced the research status of cancer-associated fibroblasts-related lncRNAs in OSCC metastasis and discussed the research prospects of lncRNAs-mediated crosstalk between OSCC cells and the tumor microenvironment in OSCC metastasis, especially the potential research value of exosomes and lymphangiogenesis, aiming to provide innovative ideas for research related to OSCC metastasis.

## The Role of LncRNAs in Cancer Metastasis

Studies have shown that lncRNAs can affect tumor metastasis by regulating a variety of biological processes, such as chromatin modification (18), transcription activation (18, 25), transcription interference (26), RNA stability regulation (15), and mRNA translation (16), etc. Xu et al. found that lncRNA SATB2-AS1 (antisense transcript of special AT-rich binding protein 2) is lowly expressed in colorectal cancer (CRC) tissues. Further research showed that SATB2-AS1 could directly bind to WDR5 (WD repeat domain 5) and GADD45A (growth arrest and DNA damage-inducible alpha), and then activate SATB2 transcription by mediating the tri-methylation of lysine 4 on histone 3 (H3K4me3) and the DNA demethylation of the promoter region of SATB2, thus inhibiting the migration, invasion, and metastasis of CRC (18). In contrast, lncRNA CF129 (CF129145.1) was confirmed to inhibit the invasion and metastasis of pancreatic cancer cells by interfering with the transcription of FOXC2 (forkhead box protein C2) (26). Intriguingly, lncRNA BLACAT2 (bladder cancer-associated transcript 2) is also able to bind directly to WDR5, but it promotes lymphangiogenesis and lymphatic metastasis of bladder cancer by enhancing the expression of VEGF-C (vascular endothelial growth factor C) (17). Additionally, He et al. reported the inhibitory effect of long intergenic noncoding RNA 1093 (LINC01093) on the migration, invasion, and metastasis of liver cancer cells. Mechanistically, by directly binding to IGF2BP1 (insulin-like growth factor 2 mRNA-binding protein 1), LINC01093 could interfere with the interaction between IGF2BP1 and GLI1 (glioma-associated oncogene homolog 1) mRNA, thus leading to the degradation of GLI1 mRNA, which further affects the expression of GLI1 downstream genes involved in the development of liver cancer (15). In addition, in breast cancer, lncRNA RP1 (RP1-5O6.5) was demonstrated to promote the invasion and metastasis of tumor cells through inhibiting the translation of p27kip1 mRNA (16).

Notably, lncRNAs can function as competitive endogenous RNAs (ceRNAs) to sponge miRNAs, blocking their regulation on the expression of their downstream genes, and then inducing various biological effects (27). Moreover, functioning as ceRNAs is also an important mechanism by which lncRNAs regulate tumor metastasis. For example, *via* competitively binding to miR-16-5p, lncRNA AGAP2-AS1 (AGAP2 antisense RNA 1) could weaken the inhibitory effect of miR-16-5p on annexin A11, thereby promoting the migration and invasion of liver cancer cells by enhancing the activation of AKT (protein kinase B) signal pathway (28). Similarly, lncRNA JPX (just proximal to Xist) was indicated to directly target miR-33a-5p, thus upregulating the expression of its downstream gene Twist1, which in turn promotes the metastasis of lung cancer cells through activating the Wnt/β-catenin signaling pathway (29).

In general, metastasis is a common feature in the development of many tumors, and the mechanisms of metastasis in different tumors are similar to some extent (30). Therefore, lncRNAs may also regulate the migration, invasion, and lymphangiogenesis of OSCC *via* (but not limited to) the above mechanisms, thereby inhibiting or promoting the metastasis of OSCC (**Table 1**).

**Table 1:** Regulatory roles of long noncoding RNAs (lncRNAs) in the metastasis-related biological processes of oral squamous cell carcinoma (OSCC).

LncRNA	Expression in OSCC	Correlation with tumor metastasis	Functions in OSCC	Potential mechanism	Refs
H19	Overexpressed	Positive	↑Migration, invasion, EMT	CeRNA (let-7a/HMGA2 axis; miR-138/EZH2 axis)	(31, 32)
LINC00152	Overexpressed	Positive	↑Migration, invasion, EMT	CeRNA (miR-139-5p; miRNA-193b-3p/PI3K/AKT axis)	(33, 34)
HOXA11-AS	Overexpressed	–	↑Migration, invasion, EMT	CeRNA (miR-98-5p/YBX2 axis)	(35)
GAS5	Downregulated	–	↓Migration, invasion, EMT	CeRNA (miR-21/PTEN/PI3K/AKT axis)	(36)
MALAT1	Overexpressed	Positive	↑Migration, invasion, EMT	CeRNA (miR-140-5p/PAK1 axis; miR-124/JAG1 axis); AKT pathway; Wnt/β-catenin pathway; NF-κB pathway	(21, 37) (38) (39) (40)
HOTAIR	Overexpressed	Positive	↑Migration, invasion, EMT	CeRNA (miR-326/MTA2 axis); Chromatin modification; SNPs	(41, 42) (43) (44)
MEG3	Downregulated	Negative	↓Migration, invasion	CeRNA (miR-548d-3p/SOCS5-SOCS6/JAK/STAT axis; miR-21); Wnt/β-catenin pathway	(45) (24, 46)
AFAP1-AS1	Overexpressed	–	↑Migration, invasion, EMT	Wnt/β-catenin pathway	(23)
UCA1	Overexpressed	Positive	↑Migration, invasion, EMT	CeRNA (miR-124/TGF-β1/JAG1/Notch axis; miR-143-3p/MYO6 axis); Wnt/β-catenin pathway	(47) (22, 48)
TUG1	Overexpressed	Positive	↑Migration, invasion	CeRNA (miR-524-5p/DLX1 axis; miR-219/FMNL2 axis); Wnt/β-catenin pathway	(49) (50, 51)
CASC15	Overexpressed	–	↑Migration, invasion	CeRNA (miR-33a-5p/ZEB1 axis)	(52)
Lnc-p23154	Overexpressed	Positive	↑Migration, invasion, EMT	CeRNA (miR-378a-3p/Glut1 axis)	(53)
CCAT1	Overexpressed	–	↑Migration, invasion	CeRNA (miR-181a/Wnt/β-catenin axis); AKT pathway	(54) (55)
LINC01116	Overexpressed	Positive	↑Migration, invasion, EMT	CeRNA (miR-136/FN1 axis)	(56)
SNHG17	Overexpressed	Positive	↑Migration, invasion	CeRNA (miR-876/SP1 axis)	(57)
SNHG16	Overexpressed	–	↑Migration, invasion, EMT	–	(58)
SNHG6	Overexpressed	Positive	↑EMT	–	(59)
SNHG3	Overexpressed	–	↑Migration	By recruiting ELAVL1 to stabilize NFYC mRNA	(60)
AC132217.4	Overexpressed	Positive	↑Migration, EMT	By upregulating IGF2	(61)
LINC00460	Overexpressed	Positive	↑Migration, invasion, EMT	By promoting PRDX1 to enter the nucleus	(62)
HNF1A-AS1	Overexpressed	Positive	↑Migration, EMT	Notch pathway	(63)
FTH1P3	Overexpressed	–	↑Migration, invasion	AKT pathway	(64)
MIR31HG	Overexpressed	Positive	↑Migration, invasion	By facilitating the formation of HIF-1 complex	(65)
HAS2-AS1	Overexpressed	Positive	↑Invasion, EMT	By enhancing the expression and stability of HAS2	(66)
HOTTIP	Overexpressed	Positive	↑Migration, invasion, EMT	CeRNA (miR-124-3p/HMGA2/Wnt/β-catenin axis)	(67, 68)
LEF1-AS1	Overexpressed	–	↑Migration	Hippo pathway	(69)
CCAT2	Overexpressed	Positive	↑Migration, invasion	–	(70)
PANDAR	Overexpressed	Positive	↑Migration, invasion	–	(71)
NKILA	Downregulated	–	↓Migration, invasion	NF-κB pathway	(72)
RC3H2	Overexpressed	Positive	↑Migration, invasion	CeRNA (miR-101-3p/EZH2 axis)	(73)
TIRY	Overexpressed	–	↑Migration, invasion, EMT	CeRNA (miR-14/Wnt/β-catenin axis) based on exosomes	(74)

## Overview of LncRNAs Involved in OSCC Metastasis

### LncRNAs Regulate Metastasis by Inducing EMT

Epithelial-mesenchymal transition (EMT) is a process of epithelial phenotype cells differentiating into mesenchymal phenotype cells. In this process, epithelial cells lose polarity and intercellular adhesion and instead acquire the characteristics of migration and invasion (75). Furthermore, the expression levels of some EMT markers will also change significantly during this process, mainly manifested by the decreased expression of E-cadherin (76) and Claudin1 (77), as well as the increased expression of β-catenin (78), Snail (79) and Vimentin (76), etc. Studies have shown that some lncRNAs regulate the metastasis of OSCC by inducing or inhibiting the EMT process. HOX transcript antisense intergenic RNA (HOTAIR) is a typical example of carcinogenic lncRNAs, which has been found to play a key role in the metastasis of various tumors. For example, in gastric cancer, HOTAIR, functioning as a connecting scaffold, could facilitate the ubiquitination and degradation of Runx3 (runt-related transcription factor 3) by enhancing the interaction between E3 ubiquitin ligase Mex3b (mex-3 RNA binding family member B) and Runx3, thus inhibiting the expression of E-cadherin and Claudin1, and ultimately promoting the migration and invasion of gastric cancer (80). Specifically, HOTAIR has also been shown to be involved in the regulation of OSCC metastasis. Lu et al. revealed that HOTAIR is highly expressed in OSCC tissues, especially in tissues with metastasis. Subsequent studies showed that overexpression of HOTAIR could enhance the metastatic potential of OSCC cells and the expression of EMT markers such as Vimentin, Snail, and Twist. It is worth noting that in clinical samples, HOTAIR is positively correlated with the expression of mesenchymal markers (Snail, Vimentin) and negatively correlated with the expression of epithelial marker (E-cadherin) (41). Additionally, Wu et al.’s research also yielded similar results. Their data indicated that HOTAIR overexpression is closely related to OSCC metastasis. Moreover, the overexpression of HOTAIR points to lower overall survival and disease-free survival. Mechanistically, HOTAIR is able to recruit histone lysine methyltransferase EZH2 (enhancer of zeste homolog 2) to the promoter region of E-cadherin and then promote H3K27me3 (tri-methylation of lysine 27 on histone 3), thus downregulating the expression of E-cadherin. These data suggest that HOTAIR may regulate the metastasis of OSCC cells by promoting their EMT (42).

In addition to HOTAIR, lncRNA SNHG16 (small nucleolar RNA host gene 16) is also overexpressed in OSCC tissues and can promote the migration and invasion of OSCC cells. Mechanism studies showed that silencing SNHG16 will increase the expression of E-cadherin, and also downregulate the expression of N-cadherin and Snail (58). Interestingly, another lncRNA of the SNHG family, SNHG6 (small nucleolar RNA host gene 6), has also been confirmed to be highly expressed in OSCC tissues, and the expression level of SNHG6 is closely related to lymph node metastasis. Meanwhile, the knockdown of SNHG6 could induce the expression of E-cadherin and inhibit the expression of N-cadherin and Vimentin (59). HNF1A-AS1 (HNF1A antisense RNA 1) is another lncRNA that is highly expressed in OSCC tissues and can promote tumor cell migration and EMT. Mechanistically, HNF1A-AS1 exerts its pro-metastatic effect by activating the Notch signaling pathway (63). In highly metastatic OSCC cells, Li et al. found that the expression of lncRNA AC132217.4 is significantly upregulated. Not only that, but AC132217.4 could also promote the migration and EMT of OSCC cells and accelerate its metastasis. The mechanism is that AC132217.4 interacts with the 3’UTR (3’untranslated region) of IGF2 (insulin-like growth factor 2) mRNA and enhances its stability, thereby enhancing the expression level of IGF2 (61). In another experiment, LINC00460 (long intergenic noncoding RNA 460) was also observed to be highly expressed in OSCC tissues and cells, and its expression level is positively correlated with lymph node metastasis. Further research exhibited the promotion of LINC00460 on the migration, invasion, EMT, and metastasis of OSCC cells. Mechanism research confirmed that there is a direct interaction between LINC00460 and PRDX1 (peroxiredoxin 1) protein, which could facilitate PRDX1 to enter the nucleus. Subsequently, PRDX1 will be enriched in the promoter regions of EMT-related genes, such as ZEB1 (zinc finger E box binding homeobox 1), ZEB2 (zinc finger E box binding homeobox 2), and Vimentin, and plays a role in promoting transcription (62). In a nutshell, EMT is a key driving factor for OSCC metastasis, and lncRNAs also play an important role in the EMT of OSCC cells. However, more research is still needed to better understand the mechanisms by which lncRNAs regulate the EMT of OSCC cells.

### LncRNAs Regulate Metastasis Through the Wnt/β-Catenin Signaling Pathway

Wnt/β-catenin signaling pathway is one of the key pathways regulating tumor development. To date, activation of the Wnt/β-catenin signaling pathway has been proved to contribute to the metastasis of lung cancer (81), CRC (82), breast cancer (83), and OSCC (39, 45). Studies have shown that lncRNAs are important factors in regulating the Wnt/β-catenin signaling pathway of OSCC cells. Liu et al. reported that MEG3 is able to inhibit the migration of OSCC cells by weakening the activation of the Wnt/β-catenin pathway (45). On the contrary, MALAT1 induces the migration, invasion, and EMT of tongue squamous cell carcinoma (TSCC) cells by activating the Wnt/β-catenin pathway (39). As another lncRNA that is highly expressed in OSCC tissue, the expression level of AFAP1-AS1 is negatively correlated with overall survival. Further research confirmed that AFAP1-AS1 is capable of promoting the migration and invasion of OSCC by enhancing the activation of the Wnt/β-catenin pathway and the expression of EMT-related genes (23). Yang et al. validated the association between high expression of UCA1 and lymph node metastasis of OSCC. Mechanism research verified that UCA1 could boost the activation of the Wnt/β-catenin pathway, thereby promoting the metastasis of OSCC (47). Similarly, the study by Liang et al. showed that high expression of lncRNA TUG1 (taurine upregulated gene 1) is also significantly associated with lymph node metastasis of OSCC. Furthermore, knocking down TUG1 inhibits the expression of β-catenin and the invasion ability of tumor cells. However, the use of LiCl (lithium chloride), a catalyst of the Wnt/β-catenin pathway, will weaken the inhibitory effect of TUG1 knockdown on the invasion of OSCC. These data suggest that TUG1 may regulate the migration of OSCC through the Wnt/β-catenin pathway (49). Nowadays, specific mechanisms by which lncRNAs regulate the Wnt//β-catenin signaling pathway remains to be explored.

### LncRNAs Regulate Metastasis Through the AKT Signaling Pathway

AKT signaling pathway is another pathway that has been widely studied and plays an important role in the metastasis of pancreatic cancer (84), gastric cancer (85), lung cancer (86), and OSCC (38, 55). In OSCC cells, the AKT signaling pathway is also regulated by various lncRNAs. FTH1P3 (ferritin heavy chain 1 pseudogene 3) is a lncRNA that is significantly overexpressed in OSCC tissues. Knocking down FTH1P3 will inhibit the migration and invasion of OSCC cells. Further investigation showed that silencing FTH1P3 contributes to the inhibition of AKT phosphorylation, which may be the mechanism by which FTH1P3 regulates the progression of OSCC (64). Based on the analysis of the expression level of MALAT1 in tissue samples, Yuan et al. reported that the overall survival of TSCC patients with high expression of MALAT1 is significantly lower than that of patients with low expression of MALAT1. Moreover, knocking down MALAT1 will cause the phosphorylation of AKT in TSCC cells to decrease, and also significantly suppress the migration and invasion of TSCC cells, while the application of PI3K (phosphatidylinositol 3-kinase) activator could reverse the inhibitory effect of MALAT1 knockdown on the migration and invasion of TSCC cells, suggesting that MALAT1 may promote the metastasis of TSCC by activating the PI3K/AKT pathway (38). In another experiment, lncRNA CCAT1 (colon cancer-associated transcript 1) was demonstrated to be overexpressed in OSCC cells and be able to promote their migration and invasion. Further mechanistic studies identified that CCAT1 is capable of improving the expression of DDR2 (discoidin domain receptor 2) and directly interacting with it. Subsequently, DDR2 could activate the ERK (extracellular signal-regulated kinase 1)/AKT signaling pathway, thus promoting the invasion and migration of OSCC cells (55). At present, the regulatory role of lncRNAs on the AKT signaling pathway remains to be further explored.

### LncRNAs Regulate Metastasis Through the NF-κB Signaling Pathway

The key role of the NF-κB signaling pathway in tumor metastasis has been proved by several studies (87, 88). Besides, lncRNAs play an important role in the regulation of the NF-κB signaling pathway. For example, Zheng et al. found that the decreased expression of lncRNA KRT19P3 (Keratin 19 Pseudogene 3) is associated with lymph node metastasis of gastric cancer. Mechanistically, KRT19P3 can directly bind COPS7A and then enhance the stability of COPS7A protein, which subsequently promotes the deubiquitinylation of IκBα and then inactivates the NF-κB signaling pathway, thus suppressing tumor metastasis (89). Previous studies have shown that lncRNAs can also regulate the metastasis of OSCC through the NF-κB signaling pathway. Hu et al. showed that lncRNA NKILA (NF-κB interacting lncRNA) is lowly expressed in OSCC tissues and cells, and the overexpression of NKILA could reduce the invasion and migration of tumor cells, as well as the levels of IκBα and cytoplasm-p65, which indicates that NKILA may regulate the metastasis of OSCC through inhibiting the NF-κB signaling pathway (72). In contrast, in another study, MALAT1 knockdown was revealed to significantly suppress the levels of NF-κB and the migration and invasion of OSCC cells (40). However, the mechanism of how lncRNAs regulate OSCC metastasis through the NF-κB signaling pathway is still unclear.

### LncRNAs Regulate Metastasis Through the IL-6/STAT3 Signaling Pathway

IL-6/STAT3 is an important signaling pathway related to lncRNAs-caused tumor metastasis. Su et al. reported that lncRNA UICC (upregulated in cervical cancer) is highly expressed in cervical cancer tissues, and its high expression is also related to lymph node metastasis. *In vitro* and *in vivo* experiments showed that UICC could facilitate the expression of IL-6 by combining with its promoter and directly interact with the phospho-STAT3 to improve its protein stability, thus promoting tumor growth and metastasis (90). There have been some studies showing that the IL-6/STAT3 signaling pathway plays an important role in the metastasis of OSCC (91, 92), but there is no research involving the role of lncRNAs in this process.

### LncRNAs Regulate Metastasis by Functioning as ceRNAs

#### MALAT1

MALAT1 is an evolutionarily highly conserved lncRNA, and its mature transcripts are mainly located in the nucleus, which suggests that MALAT1 may play an important role in the biological processes of cells (93). Recent studies have shown that by regulating chromatin modification (94), gene transcription (95), or acting as ceRNAs (96), MALAT1 is involved in the migration, invasion, and metastasis of tumors. Taking transcription regulation as an example, MALAT1 can recruit EZH2 to the promoter region of ABI3BP (ABI family member 3 binding protein) and then promote H3K27me3, thereby inhibiting the transcription of ABI3BP and ultimately promoting the metastasis of gallbladder cancer (95). In addition, *via* directly binding to miR-1914-3p, MALAT1 is capable of enhancing the stability of YAP (Yes-associated protein) mRNA, thus promoting the invasion and metastasis of non-small cell lung cancer (96). Recently, some studies have illustrated that MALAT1 also plays the role of ceRNAs in the regulation of OSCC metastasis. Zhu et al. observed that the expression of MALAT1 in TSCC tissues is significantly increased compared to adjacent non-cancerous tissues. Moreover, high expression of MALAT1 is also closely related to lymph node metastasis. Further experiments showed that after knocking down MALAT1 in TSCC cells, the expression of miR-140-5p is enhanced while the expression of PAK1(p21-activated kinase 1) is downregulated. Mechanism studies confirmed the promoting effects of MALAT1 on the expression of PAK1 and the migration and invasion of TSCC by targeting miR-140-5p (21). Apart from that, Zhang et al.’s research also support MALAT1’s role of ceRNAs in the regulation of OSCC metastasis. Their data revealed that MALAT1 knockdown could repress the metastasis of TSCC cells *in vivo* and *in vitro* by enhancing the expression of JAG1 (jagged 1) through the miR-124/JAG1 axis (37).

#### H19

H19 is abnormally expressed in various human cancers, such as pancreatic cancer (97), nasopharyngeal cancer (98), and lung cancer (99), and is usually associated with cancer progression, metastasis, and poor prognosis. Additionally, by acting as ceRNAs, H19 has been confirmed to regulate the migration, invasion, and metastasis of various tumors, including nasopharyngeal carcinoma (98), bladder cancer (100), and breast cancer (101). For example, in nasopharyngeal carcinoma, H19 overexpression is closely related to the poor prognosis of patients. Moreover, knocking down H19 could suppress the migration and invasion of tumor cells and delay the progression of transplanted tumor and lung metastasis in nude mice. Mechanistically, H19 upregulates the expression of its downstream proto-oncogene HRAS (HRas proto-oncogene, GTPase) by sponging miRNA let-7, thus promoting the carcinogenic activity of HRAS in nasopharyngeal carcinoma (98). Similarly, the interaction between H19 and various miRNAs is also an important mechanism for its regulation of OSCC metastasis. Kou et al. found that the expression of H19 in metastatic TSCC tissues is higher than that of non-metastatic TSCC tissues, and H19 knockdown could attenuate the migration and invasion of TSCC cells. The speculated mechanism of which is that H19 potentiates the expression of HMGA2 (high mobility group AT-hook 2), a key regulator of tumor metastasis, by targeting let-7. Conversely, inhibition of let-7a will dampen the inhibitory effect of H19 knockdown on the migration and invasion of TSCC cells (31). Another study revealed that the expression of H19 is positively correlated with the TNM stage and negatively related to overall survival. Further data implied that H19 can enhance the expression of its target gene EZH2 by sponging miR-138, thereby promoting the migration, invasion, and EMT of OSCC cells (32).

#### MEG3

Unlike MALAT1 and H19, MEG3 usually plays the role of a tumor suppressor. Numerous studies have confirmed that MEG3 is lowly expressed in various tumors, such as CRC (102), bladder cancer (103), and renal cell cancer (104), and inhibits their migration, invasion, and metastasis. Zhu et al. reported that MEG3 is downregulated in CRC tissues. In addition, overexpression of MEG3 could suppress the migration, invasion, and metastasis of tumor cells by inhibiting the expression of clusterin (102). A study on bladder cancer validated that by competing with PHLPP2 (PH domain and leucine-rich repeat protein phosphatase 2) mRNA to bind miR-27a, thereby promoting the expression of PHLPP2, MEG3 can restrain the invasion and metastasis of bladder cancer cells (103). Similarly, MEG3 can also suppress the migration and invasion of OSCC by exerting the function of ceRNAs. Tan et al. proved the low expression of MEG3 in OSCC tissues. Moreover, overexpression of MEG3 contributes to the downregulation of miR-548d-3p as well as the migration and invasion of OSCC cells. Mechanistically, the inhibitory effect of MEG3 on miR-548d-3p enhances the expression of miR-548d-3p downstream targets SOCS5 (suppressor of cytokine signaling 5) and SOCS6 (suppressor of cytokine signaling 6). Next, SOCS5/SOCS6 could repress the migration and invasion of OSCC by weakening the activation of the JAK/STAT (janus kinase/signal transducer and activator of transcription) pathway (24). Another study reported that MEG3 in OSCC tissue is negatively correlated with miR-21. Subsequent dual luciferase assay identified the direct interaction between MEG3 and miR-21. Furthermore, inhibiting the expression of miR-21 will reduce the migration ability of OSCC cells, and knocking down MEG3 could partially reverse the inhibitory effect of miR-21 downregulation on the migration of OSCC cells (46), which implies that MEG3 may dampen the migration of OSCC by targeting miR-21, but its downstream molecular mechanisms need further investigation. At the same time, more *in vivo* experiments are desired to clarify the role of MEG3 in OSCC metastasis.

#### LINC00152

LINC00152 (long intergenic noncoding RNA 152), transcribed from the region between protein-coding genes PLGLB2 (plasminogen like B2) and PLGLB1 (plasminogen like B1), is also known as CYTOR (cytoskeleton regulator RNA). Through interfering with cell signaling pathways such as PI3K/AKT (105), FSCN1 (fascin actin-bundling protein 1) (106), and Wnt/β-catenin (107, 108), LINC00152 was demonstrated to promote the invasion and metastasis of diverse tumors. At present, research on the function of LINC00152 in OSCC metastasis is also mainly focused on its role of ceRNAs. By analyzing two sets of TSCC gene expression profile data (GSE30784 and GSE9844), Yu et al. found that LINC00152 is significantly upregulated in TSCC tissues, and this result was also confirmed in fresh specimens and paraffin-embedded specimens. Further analysis showed that high expression of LINC00152 is closely related to lymph node metastasis of TSCC (109). Similarly, Li et al. validated that LINC00152 is highly expressed in OSCC tissues and cells. Moreover, knocking down LINC00152 could restrain the migration, invasion, and EMT of OSCC cells *in vitro*. Mechanistically, LINC00152 might play a role in promoting cancer by competitively binding miR-139-5p (33). Beyond that, miRNA-193b-3p is also a target of LINC00152. Further data implied that the inhibitory effect of LINC00152 on miRNA-193b-3p is responsible for the activation of the PI3K/AKT signaling pathway, which contributes to the migration and invasion of TSCC *in vitro* (34). However, more *in vivo* experiments are still needed to further illustrate the impact of LINC00152 on OSCC metastasis.

#### UCA1

UCA1 was first discovered in bladder cancer (110) and has been proved to promote the migration and invasion of bladder cancer cells (111). Not only that, but UCA1 can also regulate the metastasis of gastric cancer (112), osteosarcoma (113), and breast cancer (114), etc. Taking gastric cancer as an example, Gong et al. confirmed that UCA1 expression is abnormally increased in gastric cancer tissues, and high expression of UCA1 is significantly associated with lymph node metastasis. Additionally, overexpression of UCA1 was demonstrated to promote the migration, invasion, and metastasis of gastric cancer cells. The mechanism is that the direct interaction between UCA1 and miR-203 enhances the expression of miR-203 target ZEB2 (112). Recently, abnormal expression of UCA1 has also been found to be associated with OSCC metastasis. Fang et al. observed that UCA1 is highly expressed in TSCC tissues, and its expression level is positively correlated with lymph node metastasis (115). Similarly, Zhang et al. also identified the correlation between UCA1 overexpression and the poor prognosis (lymph node metastasis and shorter survival time) in patients with TSCC. Interestingly, in TSCC cells, UCA1 can sponge miR-124 and negatively regulate each other. Further research showed that by targeting miR-124 to increase the expression of TGF-β1 (transforming growth factor β1), UCA1 can activate the JAG1/Notch pathway, which in turn promotes invasion and EMT of TSCC cells (22). Moreover, in another experiment, UCA1 was found to competitively bind miR-143-3p with MYO6 (myosin VI), thus promoting the migration, invasion, and EMT of TSCC cells (48).

#### TUG1

TUG1 is another lncRNA that has been extensively explored. One study proved that TUG1 plays a role in promoting the metastasis of CRC *via* upregulating the expression of Twist1 (116). Apart from that, by competitively binding miR-143-5p, TUG1 could enhance the expression of HIF-1α (hypoxia inducible factor 1 subunit alpha), thereby facilitating the metastasis of osteosarcoma cells (117). Similarly, the chain reaction caused by the direct binding of TUG1 to miR-455-3p also contributes to liver cancer metastasis (118). In particular, TUG1 was unveiled to promote the metastasis of OSCC by weakening the inhibitory effect of miRNAs on their downstream targets. Liu et al. confirmed that TUG1 is highly expressed in OSCC cells, and can enhance the migration potential of OSCC cells. The mechanism may be that TUG1 competitively binds miR-524-5p with DLX1(distal-less homeobox 1) and thus improves the expression of DLX1 (50). Apart from that, Yan et al. discovered that TUG1 knockdown could suppress the migration and invasion of OSCC cells, while overexpression of TUG1 will boost the metastasis of OSCC *in vivo*. Further experiments verified that there is mutual inhibition between TUG1 and miR-219, and repression of miR-219 could reverse the inhibitory effect of TUG1 knockdown on OSCC cells. Mechanistically, by acting as a ceRNA to sponge miR-219, TUG1 can enhance the expression of miR-219 target FMNL2 (formin-like protein 2) and thereby exerting its pro-metastasis effect (51).

#### Other LncRNAs Functioning as ceRNAs in OSCC Metastasis

**Figure 1:**
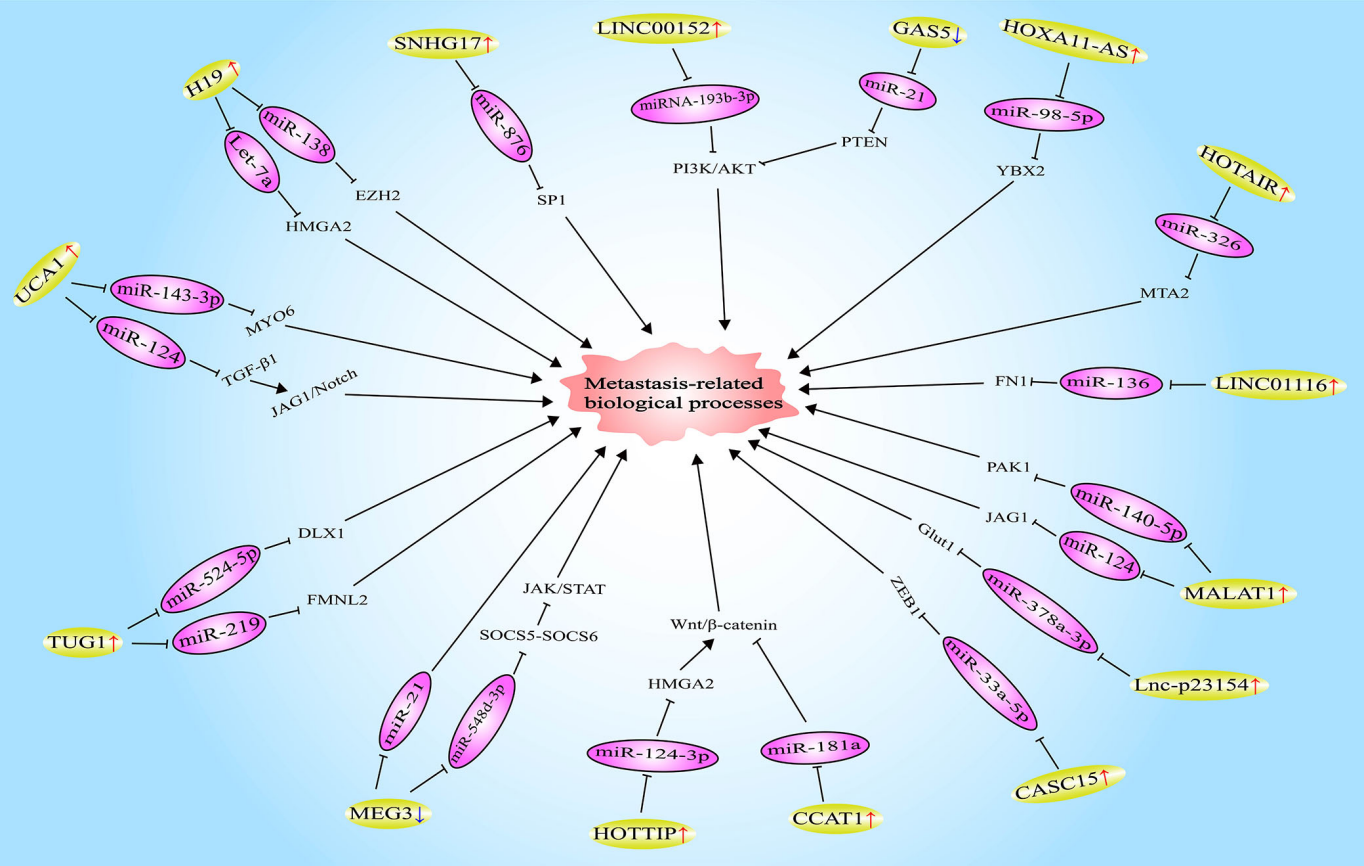
Long noncoding RNAs (LncRNAs) involved in oral squamous cell carcinoma (OSCC) metastasis by functioning as competitive endogenous RNAs (ceRNAs).

LncRNA HOXA11-AS (homeobox A11 antisense RNA) was found to be highly expressed in OSCC tissues and cells. Moreover, HOXA11-AS was demonstrated to trigger the expression of YBX2 (Y box binding protein 2) by targeting miR-98-5p, thus promoting the migration, invasion, and EMT of OSCC cells (35). In contrast, lncRNA GAS5 (growth-arrest-specific transcript 5) is lowly expressed in OSCC. Furthermore, upregulating the expression of GAS5 could inhibit the migration, invasion, and EMT of OSCC cells, and the mechanism is also to play the role of ceRNA. Specifically, GAS5 enhances the expression of PTEN (phosphatase and tensin homolog) by combining with miR-21, and then dampened the activation of the PI3K/AKT signaling pathway. Beyond that, the inhibition of miR-21 by GAS5 also increased the expression of E-cadherin, while decreased the expression of N-cadherin, Vimentin, and Snail, suggesting that the EMT of OSCC cells was repressed as well (36). The results of another study showed that HOTAIR can improve the expression of MTA2 (metastasis-associated gene 2) *via* the miR-326/MTA2 axis, thereby facilitating the migration, invasion, and EMT of OSCC cells (43). Similarly, lncRNA CASC15 (cancer susceptibility candidate 15) has also played a role in promoting metastasis in the development of OSCC. The mechanism may be that the combination of CASC15 and miR-33a-5p elevates the expression level of the downstream target ZEB1 of miR-33a-5p (52). Wang et al. found that lncRNA lnc-p23154 is highly expressed in OSCC tissues and cells. Mechanism studies confirmed that by directly binding to miR-378a-3p, lnc-p23154 is capable of upregulating the expression of Glut1 (glucose transporter 1), thereby reinforcing the glycolysis of OSCC cells, and ultimately inducing the migration, invasion, EMT, and metastasis of OSCC cells (53). Additionally, lncRNAs CCAT1 (54), LINC01116 (long intergenic noncoding RNA 1162) (56), SNHG17 (small nucleolar RNA host gene 17) (57), and RC3H2 (73) have been proved to facilitate the metastasis-related biological behaviors of OSCC cells *via* regulating miR-181a/Wnt/β-catenin, miR-136/FN1 (fibronectin1), miR-876/SP1 (specificity protein 1), and miR-101-3p/EZH2 signaling pathways respectively (**Figure 1**). In a word, lncRNAs play a key role in the metastasis of OSCC by acting as ceRNAs.

### LncRNAs Involved in the Distant Metastasis of OSCC

The probability of distant metastasis occurring in patients with OSCC is relatively low, only about 10% (119, 120). However, once a distant metastasis occurs, the patient’s hope of being cured is very slim. Studies have unveiled that lncRNAs participate in regulating the distant metastasis of various tumors including OSCC. Wang et al. found that lncRNA APC1 (adenomatous polyposis coli 1) is lowly expressed in CRC tissues, and its expression level is negatively correlated with distant metastasis. In addition, in a nude mouse model, the overexpression of APC1 could attenuate lung metastasis of CRC cells. The mechanism may be that APC1 directly binds to Rab5b mRNA and reduces its stability, thereby inhibiting the production of exosomes derived from tumor cells (121). In contrast, lncRNA BX11 (BX111887) is overexpressed in pancreatic cancer, and its expression level is positively related to distant metastasis. Further research confirmed that by recruiting the transcription factor YB1 (Y-box binding protein 1) to the promoter region of ZEB1, BX11 is able to activate the transcription of ZEB1, thus promoting the distant metastasis of tumor cells (122).

Currently, there are few studies on the role of lncRNAs in the distant metastasis of OSCC. LncRNA HOTTIP (HOXA transcript at the distal tip) was reported to be overexpressed in TSCC tissues, and its high expression is associated with distant metastasis (120). Mu et al. observed that knocking down HOTTIP could restrain the migration, invasion, and EMT of TSCC cells (67). Moreover, the results of another study identified that HOTTIP is upregulated in TSCC tissues with lymph node metastasis compared to those without lymph node metastasis. Mechanism research showed that by targeting miR-124-3p, HOTTIP is capable of enhancing the expression of HMGA2 (high mobility group AT-hook 2), which will further activate the Wnt/β-catenin pathway and promote the migration and invasion of TSCC cells (68). However, potential mechanisms of HOTTIP to regulate the distant metastasis of OSCC remain to be studied. In addition to HOTTIP, the high expression of lncRNA CCAT2 (colon cancer-associated transcript 2) (70) and lncRNA PANDAR (promoter of CDKN1A antisense DNA damage activated RNA) (71) are all associated with the distant metastasis of OSCC. Furthermore, both knockdowns of CCAT2 and PANDAR could inhibit the migration and invasion of OSCC cells. Whereas, the mechanisms by which CCAT2 and PANDAR regulate the distant metastasis of OSCC are still unclear.

### Other Potential Mechanisms of LncRNAs Regulating the Metastasis of OSCC

To evaluate the influence of HOTAIR gene polymorphism and environmental factors on the susceptibility of OSCC, Su et al. detected four single-nucleotide polymorphisms (SNPs) of HOTAIR, namely rs920778, rs1899663, rs4759314, and rs12427129, in 1200 control participants and 907 OSCC patients. They found that rs920778 is associated with a high rate of lymph node metastasis. In addition, a motif search on the HOTAIR enhancer region showed that rs920778 is located in the putative binding motif of PRDM14 (PRDI-BF1 and RIZ domain containing 14). In particular, PRDM14 is a transcription factor that plays an anti-cancer role in HPV-positive OSCC cells (123). Moreover, data from the GTEx (gnotype-tissue expression) database shows that HOTAIR is differentially expressed in muscle skeletal tissues of individuals carrying the polymorphic allele of rs920778. These results suggest that the alteration in HOTAIR expression caused by SNPs might affect the development of OSCC, and this change may come from abnormal regulation of HOTAIR expression caused by SNPs, the mechanisms of which needs to be further explored (44).

Studies have validated that in a hypoxic environment, some lncRNAs could accelerate the metastasis of OSCC. Shih et al. reported that the expression of lncRNA MIR31HG (miR-31 host gene) in OSCC tissues is increased and can be induced by hypoxia. In addition, MIR31HG overexpression is also closely related to lymph node metastasis. Furthermore, MIR31HG in the hypoxic environment has a more obvious promotion effect on the migration, invasion, and metastasis of OSCC cells, compared with that in the normoxic environment. Mechanism studies confirmed that MIR31HG is capable of promoting the transcription of HIF-1α and bind to it, thereby assisting its binding to HIF-1β (hypoxia inducible factor 1 subunit beta) and histone acetylase p300, thus forming the HIF-1 complex. Apart from that, MIR31HG is also able to recruit the HIF-1 complex to the promoter region of the metastasis-promoting genes, such as VEGF (vascular endothelial growth factor), LICAM(L1 cell adhesion molecule), and LOXL2 (lysyloxidase homologe2), to activate their transcription (65). Similarly, lncRNA HAS2-AS1 (hyaluronan synthase 2 antisense 1) is highly expressed in OSCC tissues and cells under hypoxic conditions, and HAS2-AS1 overexpression is also closely associated with lymph node metastasis. Subsequent studies exhibited that hypoxia-induced overexpression of HAS2-AS1 depends on HIF-1α, which can directly bind to the promoter region of HAS2-AS1 and activate its transcription. Additionally, HAS2-AS1 was proved to promote the hypoxia-induced invasion and EMT of OSCC cells. The potential mechanism is that HAS2-AS1 increases the level of hyaluronic acid in OSCC cells by enhancing the expression and stability of HAS2 (hyaluronan synthase 2) (66).

LncRNA SNHG3 (small nucleolar RNA host gene 3) was found to be overexpressed in OSCC cells. Silencing SNHG3 will inhibit the migration ability of OSCC cells. Further research showed that by recruiting ELAVL1 (ELAV like RNA-binding protein 1) to bind with NFYC (nuclear transcription factor Y subunit gamma) mRNA and then enhance its stability, SNHG3 could increase the expression of NFYC and thereby induce the migration of OSCC cells (60). As another lncRNA highly expressed in OSCC tissues and cells, LEF1-AS1 (lymphoid enhancer-binding factor 1 antisense RNA 1) has been validated to inhibit the activation of the Hippo pathway by direct interaction with LATS1 (large tumor suppressor 1), thus promoting the migration of OSCC cells (69). In general, mechanisms by which lncRNAs regulate OSCC metastasis are complex. One lncRNA could regulate OSCC metastasis through multiple mechanisms, while diverse lncRNAs acting on the same signaling pathway may produce opposite effects. To fully understand the biological function of lncRNAs, especially its role in OSCC metastasis, there are still many studies to be carried out.

## The Regulation of CAFs-Related LncRNAs on the Metastasis of OSCC

**Figure 2:**
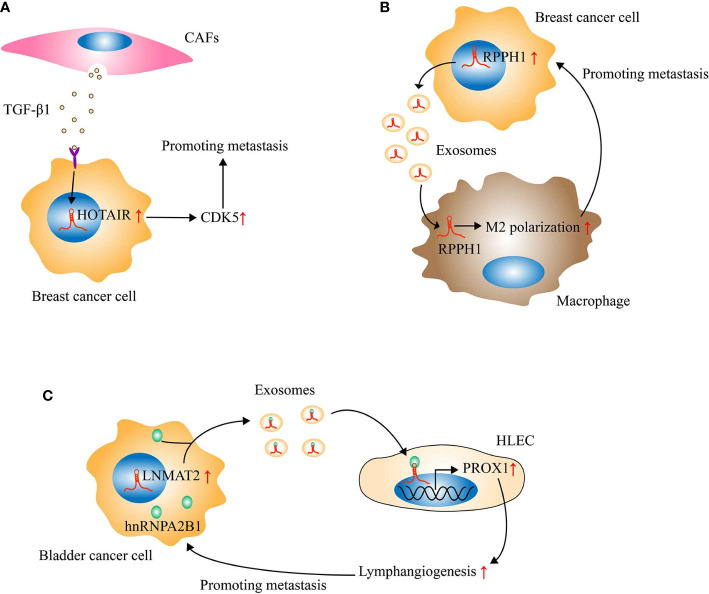
Long noncoding RNAs (LncRNAs) regulate tumor metastasis by mediating the crosstalk between tumor cells and tumor microenvironment. **(A)** Cancer-associated fibroblasts (CAFs) derived from breast cancer could secrete TGF-β1 to activate the transcription of HOX transcript antisense intergenic RNA (HOTAIR) in breast cancer cells, thereby enhancing the expression of CDK5 and then inducing metastasis. **(B)** Through exosomes, RPPH1 derived from breast cancer cells could be transported into macrophages and then induce M2 polarization of macrophages, which will further accelerate the metastasis of breast cancer. **(C)** LNMAT2 could be encapsulated into the exosomes secreted by bladder cancer cells through direct interaction with hnRNPA2B1. Subsequently, the exosomal LNMAT2 internalized by human lymphatic endothelial cell (HLEC) could promote the expression of PROX1 in HLEC, thus leading to lymphangiogenesis and lymph node metastasis.

In recent years, the tumor microenvironment composed of cells (such as fibroblasts, endothelial cells, and immune cells) and extracellular components (such as growth factors, hormones, and extracellular matrix) has become a hot spot in tumor research (124). Studies have confirmed that the heterogeneity of the tumor microenvironment contributes to the occurrence and metastasis of tumors (125, 126). Cancer-associated fibroblasts (CAFs) are an important component of the tumor microenvironment. The interaction of CAFs with tumor cells not only plays an important part in mediating the formation of CAFs (127) but also affects the process of tumor metastasis (128). It interests to note that lncRNAs participates in the regulation of CAFs on the metastasis of tumors (**Figure 2**). Ren et al. reported that high expression of HOTAIR is associated with breast cancer metastasis. In addition, the conditioned medium of CAFs-derived from breast cancer could significantly enhance the expression of HOTAIR and promote the metastasis of breast cancer cells. Subsequently, mechanism research validated the promoting effect of TGF-β1 secreted by CAFs on the transcription of HOTAIR in breast cancer cells, which will enhance the expression of CDK5 (cyclin dependent kinase 5), thereby facilitating the metastasis of breast cancer cells (129). In another experiment, lncRNA LINC00092 (long intergenic noncoding RNA 92) was found to be highly expressed in ovarian cancer tissues, and its overexpression is associated with tumor metastasis. Further investigation showed that CXCL14 (C-X-C motif chemokine ligand 14) derived from CAFs could increase the expression of LINC00092 in ovarian cancer cells. Moreover, LINC00092 was demonstrated to promote the glycolysis of tumor cells and induce the metastasis of ovarian cancer cells by enhancing the expression of glycolytic enzyme PFKFB2 (6-phosphofructo-2-kinase/fructose-2,6-bisphosphatase 2) and combining with it (130). In addition to regulating the expression of lncRNAs in tumor cells, CAFs may also regulate tumor metastasis by delivering lncRNAs to tumor cells. Ren et al. confirmed that CAFs derived from CRC are capable of transferring H19 to CRC cells through exosomes, thereby increasing the level of H19 in tumor cells and promoting the proliferation of tumor cells. The mechanism is that the direct binding of H19 to miR-141 activates the Wnt/β-catenin signaling pathway in tumor cells (131). As mentioned earlier, the activation of the Wnt/β-catenin signaling pathway has been shown to promote the metastasis of CRC (82). Besides, H19 overexpression is also an important factor inducing CRC metastasis (132). However, further research is needed to confirm whether CAFs-derived H19 can promote the metastasis of CRC.

Currently, only one study has explored the role of lncRNAs in the regulation of CAFs on the metastasis of OSCC. Jin et al. observed that lncRNA TIRY is highly expressed in CAFs derived from OSCC tissues. Furthermore, after co-cultivation of OSCC cells with conditioned medium of CAFs treated with TIRY overexpression plasmids, the capabilities of migration and invasion of OSCC cells were significantly improved. Moreover, knocking down TIRY caused increased expression of miR-14 in CAFs and their exosomes, whose mechanism is the direct binding of TIRY to miR-14. Further research showed that the overexpression of TIRY inhibits the transmission of miR-14 from CAFs to tumor cells through the transport of exosomes, thus enhancing the activation of the Wnt/β-catenin signaling pathway in tumor cells and promoting EMT. This may be the mechanism by which TIRY in CAFs regulates the migration and invasion of OSCC cells. Interestingly, the expression of miR-14 in OSCC cells was substantially increased after treatment with conditioned medium of TIRY-silenced CAFs (74). However, it is still unknown whether TIRY can be directly transported into OSCC cells through CAFs-derived exosomes and then affect the metastasis of OSCC. At present, research on the role of CAFs-related lncRNAs in OSCC metastasis has just begun, and there is still much work to be done.

## Conclusion

Research in the past decade has partially revealed the key role played by lncRNAs in the development of OSCC. In particular, abnormally expressed lncRNAs are also important factors affecting OSCC metastasis. LncRNAs can regulate the metastasis of OSCC through different mechanisms at epigenetic (18), transcription (26), and post-transcriptional (16) levels, and play the role of oncogene or tumor suppressor. Nonetheless, the current focus of scholars is mainly on the effect of abnormal expression of autologous lncRNAs in OSCC cells on tumor metastasis. The role of lncRNAs-mediated crosstalk between OSCC cells and the tumor microenvironment in tumor metastasis is still seldom studied. However, the stromal cells (such as CAFs (129), endothelial cells (133) and immune cells (134)), exosomes (135), and lymphangiogenesis (133) involved in this crosstalk have been shown to be involved in the metastasis of other tumors (**Figure 2**).

As mentioned previously, based on the crosstalk between CAFs and tumor cells, CAFs-related lncRNAs play an important role in tumor metastasis. It is worth noting that exosomes can act as messengers between tumor cells and CAFs. Moreover, exosomes are also the key link between tumor cells and other stromal cells, which contribute to the metastasis of various tumors as well (133, 134). The regulation of exosomes on tumor metastasis depends on their special structure (membranous microcapsules with diameters ranging from 50 to 150nm) and contents (RNA, DNA, proteins, etc.) (136). Previous studies have identified that exosomes could transfer lncRNAs to target cells and induce a series of chain reactions, thereby regulating tumor metastasis (134, 135). For example, Liang et al. found that the expression of lncRNA RPPH1 (ribonuclease P RNA component H1) in breast cancer tissues is significantly upregulated. Moreover, RPPH1 overexpression is associated with metastasis and poor prognosis. In addition, the *in vivo* study demonstrated that RPPH1 is able to promote the metastasis of breast cancer. Mechanistically, through exosomes transport into macrophages, RPPH1 derived from breast cancer cells could induce M2 polarization of macrophages. In turn, M2-polarized macrophages will further accelerate breast cancer metastasis (134). However, there is currently no study on the effect of exosomal lncRNAs on OSCC metastasis. Therefore, further exploration of the role of exosomal lncRNAs in OSCC metastasis will provide more valuable information on the metastasis of OSCC.

The high rate of lymph node metastasis is the main cause of death in patients with OSCC (5). Not only OSCC, but many other tumors can also have lymph node metastasis (112, 133, 134). As an important channel for lymph node metastasis of tumor cells, lymphatic vessels and the mechanisms of their generation naturally attract the attention of scholars. Studies have confirmed that lncRNAs are involved in inducing lymphangiogenesis of tumors (133, 137). Taking lncRNA LNMAT2 (lymph node metastasis-associated transcript 2) as an example (133), Chen et al. found that bladder cancer-derived exosomal LNMAT2 can enhance the ability of tube formation and migration of human lymphatic endothelial cells (HLEC) *in vitro*, and facilitate tumor lymphangiogenesis and lymph node metastasis *in vivo*. Further mechanistic studies uncovered that LNMAT2 could be encapsulated into exosomes secreted by bladder cancer cells through direct interaction with hnRNPA2B1 (heterogeneous nuclear ribonucleoprotein A2B1). Subsequently, the exosomal LNMAT2 will be internalized by HLEC, and then promote the expression of PROX1 (prospero homeobox 1) at the epigenetic level, thus leading to lymphangiogenesis and lymph node metastasis of the tumor. Therefore, LNMAT2 has the potential to serve as a therapeutic target for lymph node metastasis of bladder cancer. However, to date, there has been no research on the regulation of lncRNAs on OSCC lymphangiogenesis. Subsequent research should pay more attention to this direction.

In addition to the crosstalk between tumor cells and stromal cells, the crosstalk between tumor cells and the extracellular matrix (ECM) also plays a critical role in the metastasis of tumors. Huang et al. reported that integrin alpha 2 (ITGA2), upregulated in omental metastases of patients with ovarian cancer, can trigger tumor cell adhesion to collagen, and then facilitates the migration and peritoneal metastasis of tumor cells. The speculated mechanism of which is that ITGA2 activates the phosphorylation of focal adhesion kinase and the mitogen-activated protein kinase pathway (138). In another study, lncRNA CASC9 (cancer susceptibility 9) was revealed to be associated with the prognosis and metastasis of esophageal squamous cell carcinoma (ESCC). Besides, CASC9 knockdown could significantly inhibit the migration, invasion, and metastasis of ESCC cells. Mechanistically, CASC9 can enhance the enrichment of the transcriptional coactivator CREB-binding protein and H3K27 acetylation in the promoter of LAMC2, an upstream inducer of the integrin pathway, thus promoting its expression. Nevertheless, the impact of the crosstalk between tumor cells and ECM regulated by lncRNAs on OSCC metastasis has not been studied.

In summary, lncRNAs are expected to serve as indicators for screening, treatment, and prognostic monitoring of OSCC metastasis. However, the current understanding of the role of lncRNAs in OSCC metastasis is still in its infancy. Therefore, further research on the role of lncRNAs in the crosstalk between OSCC cells and tumor microenvironment will help enrich the blueprint of the mechanisms of OSCC metastasis.

## Author Contributions

YX and EJ discussed and designed the study. YX performed the research and analyzed the data. ZS and ZJS revised the article. All authors contributed to the article and approved the submitted version.

## Funding

This study was supported by grants from the National Natural Science Foundation of China 81772897, 81672666.

## Conflict of Interest

The authors declare that the research was conducted in the absence of any commercial or financial relationships that could be construed as a potential conflict of interest.
